# Mixed methods in evaluating acupuncture and standard care for pregnant women with back pain: ease back

**DOI:** 10.1186/1745-6215-14-S1-P96

**Published:** 2013-11-29

**Authors:** Bernadette Bartlam, Annette Bishop, Melanie Holden, Ismail Khaled, Christine Kettle, Nadine Foster

**Affiliations:** 1 Keele University, Keele, UK; 2 University of Birmingham, Birmingham, UK; 3 Staffordshire University, Stoke-on-Trent, UK

## 

EASE BACK is an NIHR HTA funded feasibility pilot trial to inform a large randomised trial evaluating the clinical and cost-effectiveness of acupuncture for pregnant women with low back pain (LBP). Mixed methods were employed to generate a breadth of data. In phase 1, interviews and focus groups explored the views of pregnant women with LBP about the acceptability of the proposed interventions, the content and delivery of participant information, important outcomes, and timing of outcome measurement; and the views of health professionals (midwives and physiotherapists) about standard care, using acupuncture for this patient group, proposed trial design, and recruitment methods. A total of 52 individuals were interviewed. In addition, a postal survey of UK based physiotherapists was undertaken (response rate 57.5%: *n*=629/1093). Integrated analysis of both methods highlighted the extensive impact of moderate-severe LBP, the paucity of effective interventions, the pivotal nature of the midwife-patient relationship, and physiotherapists’ concerns about using acupuncture for pregnant women, despite their use of it for other musculoskeletal problems. Results informed phase 2 (pilot trial) in terms of patient information, recruitment and consent procedures. They also helped shape the training programme for physiotherapists delivering the interventions, to directly address issues of safety and side effects. Findings highlight the role mixed methods can play in preparing for randomised trials and ensuring they are sensitive to the needs of patients and practitioners. The phase 2 pilot trial is currently underway.

